# The quest for choice and the need for relational care in mental health work

**DOI:** 10.1007/s11019-014-9563-z

**Published:** 2014-04-24

**Authors:** Børge Baklien, Rob Bongaardt

**Affiliations:** Faculty of Health Studies, Telemark University College, Box 203, 3901 Porsgrunn, Norway

**Keywords:** Anti-psychiatry, Consumers, R.D. Laing, Logic of care, Relational health, Service users, H.S. Sullivan, Survivors, T. Szasz

## Abstract

Since the revolutionary mood of the 1960s, patient-centered mental health care and a research emphasis on service users as experts by experience have emerged hand in hand with a view of service users as consumers. What happens to knowledge derived from firsthand experience when mental health users become experts and actively choose care? What kind of perspective do service users pursue on psychological distress? These are important questions in a field where psychiatric expertise on mental illness is socially structured and constrained as an intra-personal disturbance of the mind. We argue that experience experts have lost a coherent perspective on care and health. We illustrate this by rationally reconstructing how the interpersonal view of mental health first gained and then lost coherence between the conception of mental health, the practice of mental health care, and the user experience. Harry Stack Sullivan’s interpersonal theory was a paradigm case for such coherence. The inclusion of mental health consumers as ‘experts by experience’ in the mental health field took place at the cost of Sullivan’s coherent interpersonal theory. Service users who interact side by side with medical experts as experience experts are constrained by the evidence-based imperative and consumerism. Service users are caught up in a race among experts to gain knowledge about mental problems from a third-person perspective instead of from first-person experience. To make a contribution service users have more to gain from a research approach that appreciates that they are persons among persons rather than experts among experts.

## Introduction

During recent decades, users of mental health services have steadily increased the strength of their voice in mental health care policies, education and practice (WHO 2010). Patient-centered care, with its emphasis on the patient’s voice and choice, has become a common good in mental health care (Dahlberg et al. 2009), and service users acting as ‘experts by experience’ are pivotal in their own care and treatment plans (Pilgrim 2008).

One can wonder what happened to knowledge derived from firsthand experience when mental health service users themselves became experts and actively began to choose care. What perspectives on mental health do experts by experience endorse? The ensuing expertise is socially structured and constrained in terms of mental illness being a psychopathological intra-personal disturbance of the mind, in line with dominant psychiatric practice (Speed 2005; Bracken and Thomas 2005; Coles et al. 2013). However, alternative views on mental health are also endorsed; mental health can be seen as a relational phenomenon which is never static, in line with so-called relation-based mental health work. In such views, mental health cannot be reduced to brain activity or individual life histories, but emerges in and through processes of interaction between the person and other persons (Bracken and Thomas 2005; Crossley 2011; Frie and Coburn 2010; Kirschner and Martin 2010). In our study, we take a closer look at the expertise that users as consumers bring to the mental health care field and how their views on mental health have developed vis-à-vis the mainstream psychiatric view. In doing so, we will try to unravel the apparent paradox that experts by experience become part of the dominant medical paradigm they wished to move away from. In other words, in this study, we ask to what extent mental health users are ‘experts among experts’ at the expense of being ‘persons among persons’.

We approach these questions by generating a rational reconstruction of how the interpersonal (person among persons) view of mental health first gained and then lost coherence among its conceptions of mental health, the practice of mental health care, and the experience of the user. By rational reconstruction, we mean that it is only on the basis of what we know of user knowledge in mental health care today that we can reconstruct how this has come to be so. Moreover, we study how past participants in mental health argued and thought in order to revive their voices in the present-day context, enabling us to create a dialogue between these and currently active players in the field. We acknowledge the risk of writing whiggish history but find support in Rorty, who writes that “such enterprises in commensuration are, of course, anachronistic. But if they are conducted in full knowledge of their anachronism, they are unobjectable” (Rorty cited in Oniscik 2005, p. 245).

In the following, we first describe H.S. Sullivan’s mid-twentieth century interpersonal psychiatry, because it is a paradigmatic example of a coherent approach based on an interpersonal view of mental health. Secondly, we address how Sullivan’s coherence was lost by those whom he inspired to revolt against psychiatry, i.e., both anti-psychiatrists and service users. Thirdly, we show how a partnership between service users and mental health professionals paved the way for a shared acceptance of the breaking up of mental health into independent parameters. We discuss the consequences of a parametric view of mental health in terms of standardized mental health and the subsequent logic of choice in mental health services (Kugelmann 2003; Mol 2008). We conclude with the suggestion that a humanistic approach to mental health can allow for the voice of the service user as a person among persons rather than an expert among experts.

## Sullivan’s coherent interpersonal approach

Sullivan (1892–1949) has been credited for being the first to formulate an explicitly interpersonal theory of psychiatry, by psychiatrists as well as by service users in the so-called ‘recovery’ literature (Davidson et al. 2010; Perry 1982). He formulated an interpersonal theory of the self, mental disorder, treatment and recovery. The theory was formulated in a series of lectures during the period 1946–1947, which were published together in the book *The interpersonal theory of psychiatry* (Sullivan et al. 1953). Sullivan lived his interpersonal theory by creating a therapeutic setting in which the psychiatrist could participate in the everyday activities of the clinic. He saw himself as a person among other persons in any patient’s social world. Sullivan strongly believed that the self-contained individual with a unique individuality was an illusion. He criticized society’s focus on the individual and how the medical model of psychiatry sustained this view, referring to this as “the very mother of illusions, the ever pregnant source of preconceptions that invalidate almost all our efforts to understand other people” (Sullivan 2000/1938, p. 114). To Sullivan there is a different self in each interpersonal relationship, and the person has as many understandings of him- or herself as he or she has interpersonal relationships. The self is fundamentally social in nature and “personality is the relatively enduring pattern of recurrent interpersonal situations which characterize a human life” (Sullivan 1953, p. 110–111). The ideal of human maturity and independence leads us to believe that we are dependent on others only when we feel ill (Sullivan et al. 1970, p. 35). However, in Sullivan’s view, health and illness are both facets of living as a person among persons (Sullivan 1953, p. 313).

Sullivan’s view of mental disorder was consistent with his interpersonal view of the self. He was inspired by Meyer’s psychobiology, which endeavored to abandon a dualistic spilt between body and mind (Dowbiggin 2011), seeing them as two aspects of the same process. Like Meyer, Sullivan argued that mental illness comes from problems with living and adjustment to society. If a person is unable to adjust to or withstand the social organization in which he is embedded but experiences this organization as important, mental illness can develop (Sullivan 1953, p. 208). In this way Sullivan envisioned a continuum between mental health and illness; a person suffering from a mental illness is not fundamentally different from any other person. Hence Sullivan’s famous one-genus postulate which states that “*everyone is much more simply human than otherwise*” (Sullivan 1953, p. 32; italics in the original).

Sullivan emphasized the importance of taking part in the patient’s everyday life as a participating observer and listening to his or her point of view, which is a crucial aspect of making a social recovery. For several years he lived and worked on a daily basis among schizophrenic patients. When he was given a patient’s story by a staff member, Sullivan could refer to this story as a “wonderful works of clinical fiction” (Havens 2000, p. 129). He read first-person accounts about living in a psychiatric ward, such as the self-biographical book by Beers, a pioneer of the mental health movement of the first half of the 19th century, who underscored the relevance of the local community for any person’s mental health. Sullivan conceived of the psychiatric ward as a self-standing social world, implying psychiatric care and treatment during the other 23 h (Bloom 2002). At the psychiatric hospital, he established a one-class society and did everything to minimize the impact of his own status. He hand-picked male attendants and patients in recovery to staff the ward and taught them his “socio-psychiatric program” in which the schizophrenic patient was to partake as a “*person among persons*” (Conci 1997, p. 131; italics in the original).

With this backdrop, we can state that Sullivan had a coherent interpersonal view of the self, mental health and treatment. With hindsight, we can characterize this approach as ‘relational’, a view in which body and mind feature as an embodied self within a situation where mental health and illness are ways of experiencing and acting in situations and where mental health care takes place in a situation between two persons who meet as persons, attempting social recovery through participant observation rather than medical treatment of an alien body. In the next two sections, we aim to show how this coherence in Sullivan’s approach was lost in the work of those whom he inspired: anti-psychiatry and the user movement in mental health care.

## Anti-psychiatry: the revolt from above

Sullivan inspired the psychiatrists T. Szasz (1920–2012) and R. D. Laing (1927–1989) to develop their own ideas about mental disorders and care in a revolt against mainstream psychiatry, the so-called anti-psychiatry of the 1960s and 1970s. Crossley (1998) describes their work as a “revolt from above” (p. 878), implying a skeptical stance towards psychiatry as an instance of power and social control. Anti-psychiatry was never a broad revolutionary movement with the intent to demolish the mental health field, but rather an attempt to promote a new paradigm to normalize madness by placing behavior in an interpersonal context rather than an allegedly narrow, normative context that sustains a controlling, medical, and objectifying gaze at patients. The anti-psychiatrists argued that symptoms of mental illness are easier to understand if context is taken into account.

Sullivan’s approach impressed Szasz (2010, p. 220) with his portrayal of disease as “problems in living”. Though skeptical of the anti-psychiatry label, Szasz famously held that mental illness is a myth. Mind is not brain, he argued; behavior cannot be a disease and is not detectable as a physical defect within the individual. Phenomena studied by psychiatrists, such as racism, suicide, and murder, cannot be revealed through studies of the brain. In his view, the psychiatric establishment exercised negative power over psychiatric patients by depriving them of the rights to self-determination and freedom and the right to be regarded as citizens who can take the consequences of their own actions. Seen in this light, psychiatry is “an ideology and a technology for the radical remaking of man” (Szasz 1973, p. 11).

Szasz maintained that the medical model was not suitable to help people with their personal, social and ethical problems with living (Szasz 2010, p. 262). Hardship for modern man derives “from stresses and strains inherent in the social intercourse of complex human personalities” (Szasz 1973, p. 14). Difficulties in human relations must be analyzed and given meaning merely within specific social and ethical contexts. Problems of living, as Szasz (1973, p. 22) contended, are due to man’s awareness of himself in an expanding world and the increasing “burden of understanding”; however, modernity and man’s increasing knowledge do not take away the individual’s responsibility for his or her actions by hiding it in the notion of mental illness. Szasz gave his patients the choice whether to work or not. The therapist is not responsible for the patient’s actions, only the patient him- or herself, and any preoccupation with the therapist is a way of not attending to one’s own life. Szasz, as the libertine he was, encouraged his patients to assume responsibility and develop a self that was independent of the therapist as a way out of their problems with living.

Laing was another important anti-psychiatrist (Crossley 1998). He concurs with Sullivan’s well-known assertion that the psychotic person is primarily “simply human” (Laing 2010, p. 34). Human beings may exist in isolation but are first and foremost intimately related to other persons and the world. It may be in one’s own family that mental illness first emerges. Problems of living arise when the person is placed in a situation of conflicting expectations from family members (Laing and Esterson 1970). For Laing, mental illness was a normal response to a mad world and thereby socially intelligible (op cit. p. 27). Actions are embedded in meaningful contexts, not controlled settings, and therefore, Laing argued, existential problems can arise that are beyond the person’s control. When lacking a solid foundation of existence, the person can experience an ontological insecurity and become “preoccupied with preserving rather than gratifying himself” (Laing 2010, p. 42). An ontologically insecure individual dreads engulfment by the other, implosion into complete emptiness and transformation into a dead thing (Laing 2010, p. 43). When failing to come up with a unique voice, the person may experience a lack of autonomy and become intertwined with the other.

To Laing, people with so-called mental illness are trying to find their way back; psychotic episodes are understandable as an attempt to communicate worries and concerns, often in situations where this was not possible or not permitted. They should thus be seen as “self-acting agents”, responsible and capable of choice (Laing 2010, p. 22). In Laing’s view, the person needs help to cultivate such independence; accordingly he set up therapeutic communities—the most famous being Kinsley Hall—with the aim to support the person through his or her own voyage.

Szasz and Laing both see the psychiatric patient as a person—more human than anything else—having problems with living. Mental suffering develops within a network of social relationships. Thus far, they follow Sullivan’s relational thinking. However, where Sullivan followed through with a relational view of the self and mental health care, Szasz and Laing emphasize an independent self as the driving force to break out of unhealthy social relationships (Bracken and Thomas 2005). More often than not, human relationships were the problem, and becoming a ‘self-standing’ individual was the solution. Szasz proclaimed individual liberty and independence; persons can make their own choices, solve their own problems and are responsible for their actions. Laing suggested ways that the person might cultivate a more independent self and urged for freedom and subjectivity. Thus, they generated the confusion of offering interpersonal care to an autonomous self. Hence, the anti-psychiatrists, through the voices of Szasz and Laing, did not maintain the coherent relational view of self, mental disorder, and mental health care that had typified Sullivan.

## The mental health service user movement: the revolt from below

While anti-psychiatry revolted from above, at the delivering end of mental health care, those at the receiving end of psychiatry also let their voices be heard, initiating a revolt from below (Crossley 1998, p. 878). Judi Chamberlin (1944–2010), an active leader in the user movement, notes that users of mental health services considered anti-psychiatry largely an intellectual exercise of academics with little willingness to reach out to struggling ex-patients and their perspectives (Chamberlin 1990). Initially, user movements may have been nurtured by the anti-psychiatry movement’s critique of psychiatry’s standard medical model and the everyday life it generated in the psychiatric ward, providing a joint platform for individuals who had expressed similar criticism, such as Beers (1908) and Packard (cf. Dowbiggin 2011). However, user groups were also eager to create an identity formulated in positive terms, separate from anti-psychiatry. The user movements merged with a larger class struggle, the ‘counter-culture’ of the 1960s, which provided impetus and legitimacy (Reaume 2002). The user movement mushroomed into many different user organizations with different agendas and ideologies, and even controversy among themselves, but with a shared belief in the rights to interpret their own experiences of mental disorder, to self-determination and to help on their own terms (Barnes and Cotterell 2012).

The first-person singular voice of a few patients in the late nineteenth and early twentieth centuries had thereby gradually developed into a first-person plural identity as ‘patients/users’, ‘consumers’, or ‘survivors’ (McLean 2010; Pilgrim 2005). Through narratives from former patients, an entirely new form of self-awareness based on common experiences emerged. Service users went from formalized ascribed roles to a growing self-consciousness and self-confidence that was anchored in their unique understanding of their own illness. They did not subscribe to the anti-psychiatric claim that mental illness is not a disease but rather confirmed its status by becoming the new experts on mental illness. As experience experts side by side with medical experts, the majority of user organizations have now turned from an anti-establishment movement into a consumer coalition (Rissmiller and Rissmiller 2006). As consumers, mental health service users have acquired much of the equality they aspired to through grass-roots lobbying rather than by radical revolt and have made “peace with the mental health establishment [with] no sympathy for Szasz’s theory that mental health is a myth” (Dowbiggin 2011, p. 168).

The improvement in equality between service user and provider is based on a shared understanding of mental illness as a medical disease, i.e., as an individual’s ‘bad genes’ or ‘broken brain’ (Adame and Knudson 2008). That is to say that while the user movement strengthened the user voice, a Sullivan-like interpersonal conception of mental health was weakened in favor of a intrapersonal conception of mental health; it is only such a conception that allows one to acquire and express experiential expertise.

## Standardized versus relational health

Why are the voices of experts by experience unable to give us a coherent perspective on care and health? To explore this question we use Kugelmann’s (2003) distinction between “standardized health” and “being healthy.” According to Kugelmann (2003), standardized health appears when norms and risk factors are determined by biomedicine, and biomedicine intersects with an economic definition of wellbeing. Parameters of health are turned into medical and economic parameters. Lived experiences are turned into third-person objective categories of sickness and are thus not the health of any particular person; given are statistical averages in the intersection of biomedicine and economy. Science and health care are increasingly organized like businesses (Gadamer 1996).

Transposed to mental health, standardized mental health can be compared with a Procrustean bed that reduces and deforms its object. The first-person narratives of relational experience of distress, suffering and healing are made into parts of value-free parameters by health care providers and patients. These parameters influence service users’ behavior in such a way that they identify themselves with constructed labels even though all they may have in common is the wide range of parameters used to categorize them. They become exposed “to the anonymity of the clinical apparatus” (Gadamer 1996, p. 20). Service users are caught in a double bind. They become dependent on the classifications of diseases to attain support because the socially significant outcome that determines different forms of interventions is what constitutes sickness (Young 1982). To put it differently, the sickness does not define the intervention as much as the intervention defines the sickness. Mental health work materializes as a series of direct relational interventions to standardize these parameters. However, what happens when service users claim to be experts on their disease? The immediate grasp of the life world disappears the moment the service user raises a question from an expert perspective or third person perspective about his or her disease. The life world is no longer experienced but considered as if in the third-person plural. In other words, *they* become part of the problem of the reification of mental health.

In the emerging partnership, service users become part of the family of professionals who, by their ‘expertise of subjectivity’, can classify, measure, diagnose and prescribe remedies for psychologic illnesses (Rose 1989). Users as experience experts are trapped in a race to keep up with professional experts, as they do their own research, write their stories, campaign politically, etc. Accordingly, the more users invest in becoming experts among experts, the farther they become removed from the relational ‘persons among persons’ view of mental health work. In racing ‘upwards’ to become experts, they are captured in what Hacking (1999) termed the ‘looping effect’, where conceptual labels of mental health evoke new behaviors and where original behaviors may give rise to new labels. It implies that help-seekers’ descriptions of their experience of mental health interact with the professional knowledge that categorizes them in the first place (Dehue 2009). This profoundly illuminates the idea that when service user expertise stands on equal footing with research results and professional judgment, user experience is a moot contribution to evidence-based treatment and social support.

To sustain an alternative to the intrapersonal conception of mental health, it has been argued that user groups need to return to their roots (Lakeman et al. 2007). They need an approach where the person is portrayed in terms of lived experience; one cannot portray a person purely from outside as a “mere object of biological, psychological or sociological investigation” (Merleau-Ponty 2002, p. ix). The person and the world belong together and are inseparable; the interpersonal aspect of illness cannot be separated from the person’s lived experience (Van den Berg 1972). An alternative is required where standardized mental health is replaced by perspectives that capture the experience of being mentally healthy.

According to Kugelmann (2003), being healthy is a relational experience; one is not separated from lived experience but rather effortlessly engaged with others in situations in the world. He adopts Gadamer’s (1996) view that health is not “something that can simply be made or produced” (p. vii) … “it is a condition of being involved … being together with one’s fellow human beings” (p. 113). In this perspective, being mentally healthy involves becoming engaged in something—caring about something meaningful, for oneself, for family and friends. Such a perspective on mental health rests on a relational view of the person, and should cohere with a relational view of care.

## Logic of choice versus logic of care

The preoccupation with user voice and choice is grounded in a logic that enforces a split between the person and the world. To make this point we use Mol’s (2008) distinction between the “logic of choice” and the “logic of care.” In the logic of choice, professionals are preoccupied with what patients want and opt for. The autonomous patient’s right to choose dictates healthcare. The role of professionals is then to provide users with care options to choose from. In the logic of care, by contrast, professionals and patients are not concerned with choice but with what one actually does. In this logic, one’s ability to act depends on others in a continuing process that “goes on and on until the day you die” (Mol 2008, p. 62).

In the *pas de deux* between service users and professionals, good treatment is realized when the service user’s voice is heard and when mental health workers emphasize the service user’s choices. More choice is regarded as the road to better mental health, and it is logical to think that if some choice is good, more is better. Schwartz (2004) argues, however, that in a psychological sense this assumption may be wrong. Although some choice is undoubtedly better than none, more is not always better than less in the caring industry. Too many choices can raise your expectations to an unrealistic level, and it can make you blame yourself for any failures. You can blame yourself for not making a choice that is more or less per impossible. However, the options of choice differ with one’s situation in the world. A good swimmer and a person who cannot swim well do not have the same experience of autonomy when choosing whether to save a drowning man (Merleau-Ponty 2002, p. 508).

In the logic of choice, the individual is disengaged from the world. The world materializes as a conglomerate of neutral objects or facts that are detached from the individual. The individual engaged in the world is replaced by an individual looking at the world as sheer facts from a third-person perspective, and in this way “gives up living” (Merleau-Ponty 1964, p. 159) in it, failing to see that we “live in it from the inside” (p. 178). However, in the lived world of care, we cannot simply separate subject from object or values from facts as these are internally related.

Mol (2008) argues that care has little to do with separate individuality and user choice because care is attuned to people with respect to their basic relationship to one another. Mol puts forward an alternative logic of care where the self emerges in interpersonal situations in everyday living. In the logic of care, individuals are not ensnared in causal chains. Care is always situational, and it does not make an unbridgeable distinction between fact and value. Facts are not just neutral facts; in the logic of care, one attends to facts and values jointly (Mol 2008). In the context of care, knowledge is not information to provide a better map of reality to gradually increase certainty before risk but to gather knowledge is a way “of crafting more bearable ways of living *with*, or *in* reality” (p. 46; italics in the original). Care cannot be separated from experience, and therefore care aims to improve the situation that “meddles with every detail of our daily lives” (Mol 2008, p. 37). Care appears as tinkering, dealing with the messiness and quirkiness of everyday life, in our relationships with the world, others, and self, over time. In the relational perspective, care is not receiving care, or choosing care, but engaging in care with professionals as much as oneself in an environment which may facilitate or hamper it.

In Mol’s analysis of the logic of choice, the collective user voice is formed by individuals who are facing similar problems in living, share a diagnosis, or get the same type of treatment. The user voice acquires strength by adding more individual, autonomous voices. The users influence the market as consumers, and as citizens the sum of their votes may bring about democratic decisions. However, in the logic of care, the collective aspect forms the starting point of reasoning. The person is embedded in the family tradition, or is immersed in the work climate, with all of the habits and customs involved. In this view, the community is not one of ‘added equals’, but of entangled roles in a relational network, where not everybody is characterized by the same health- or illness-related variable. Yet, within the network, mental health can become the focal point at which the actions of persons in the network are directed. The person and his or her mental health are not characterized by open choices, but rather by living as making a plethora of small adjustments in a network of relationships to improve one’s existence.

## Concluding remarks

The current demand by mental health service users for a stronger voice and more choice is a positive development because users then take center stage in the practice of care. Service users are driven to seek care and treatment within the system of knowledge they once denied but that defines and negotiates their life world by standardizing values. Those service users who strive for equality between users and providers of care have the opportunity to contribute to care and treatment on a par with providers, insofar as this is practically possible. However, this implies that service users who become experts among experts maintain and uphold the pivotal contribution of care providers.

While users have acquired an influential presence as experts among experts, they also have gradually distanced themselves from being persons among persons. We suggest that instead of users climbing ‘upwards’ to become experts, research should reach ‘downwards’ and focus more on mental health as a relational ‘persons among persons’ phenomenon.

Mental health emerges as a lived relational phenomenon between I-oriented persons among persons, whereas it emerges as a reified object when discussed among They-oriented experts. These two different views on mental health are endorsed by different treatment and care practices but also by different systems of knowledge. Those who take a relational perspective on mental health argue that mental health emerges continually in time, in the world, in the body and in relationships with significant others and cannot be objectified to standardized parameters within an autonomous self (Crossley 2011; Frie and Coburn 2010; Martin et al. 2010). Such a relational understanding of mental health requires a human scientific approach that is “in tune with the essence of human beings” (Giorgi 1985, p. 20), a science that is close to practice and anchored in life worlds (Todres et al. 2007, Dahlberg et al. 2009).

The choice of a human scientific approach to mental health implies criticism of powerful governmental demands for evidence-based mental health care in which biomedical access to the patient’s experiences of illness makes the management of human beings become all the more powerful and coercive (Taussig 1980). However, it is also a critique of those service users who strive to become experts among experts and start to see themselves in a medical and perhaps even economic light. McKnight (1995) argues in his book “The Careless Society” that “revolutions begin when people who are defined as problems achieve the power to redefine the problem” (p. 16). The spark of a revolution from above in the anti-psychiatric critique of the legitimacy of experts’ power to define others disappeared when the revolt from below ebbed out into a consumer movement with service users who were eager to collaborate. What we need is a new service user movement grounded outside reductionist standardized health in a logic that encourages a new understanding of what experience of life is as a person among persons.
